# Dynamic histone modification signatures coordinate developmental programs in strawberry fruit ripening

**DOI:** 10.1093/hr/uhae158

**Published:** 2024-06-07

**Authors:** Qinwei Pan, Suping Guo, Jing Ding, Yue Zhou, Xiaorong Huang, Qi Qi, Feng Wang, Han Wu, Yi Li, Tingting Gu

**Affiliations:** National Key Laboratory of Crop Genetics & Germplasm Enhancement and Utilization, Nanjing Agricultural University, Nanjing 210095, `China; College of Horticulture, Nanjing Agricultural University, Nanjing 210095, China; National Key Laboratory of Crop Genetics & Germplasm Enhancement and Utilization, Nanjing Agricultural University, Nanjing 210095, `China; College of Horticulture, Nanjing Agricultural University, Nanjing 210095, China; National Key Laboratory of Crop Genetics & Germplasm Enhancement and Utilization, Nanjing Agricultural University, Nanjing 210095, `China; College of Horticulture, Nanjing Agricultural University, Nanjing 210095, China; Sanya Institute of Nanjing Agricultural University, Nanjing Agricultural University, Nanjing 210095, China; National Key Laboratory of Crop Genetics & Germplasm Enhancement and Utilization, Nanjing Agricultural University, Nanjing 210095, `China; College of Horticulture, Nanjing Agricultural University, Nanjing 210095, China; National Key Laboratory of Crop Genetics & Germplasm Enhancement and Utilization, Nanjing Agricultural University, Nanjing 210095, `China; College of Horticulture, Nanjing Agricultural University, Nanjing 210095, China; College of Horticulture, China Agricultural University, Beijing 100193, China; National Key Laboratory of Crop Genetics & Germplasm Enhancement and Utilization, Nanjing Agricultural University, Nanjing 210095, `China; College of Horticulture, Nanjing Agricultural University, Nanjing 210095, China; National Key Laboratory of Crop Genetics & Germplasm Enhancement and Utilization, Nanjing Agricultural University, Nanjing 210095, `China; College of Horticulture, Nanjing Agricultural University, Nanjing 210095, China; Department of Plant Science and Landscape Architecture, University of Connecticut, Storrs, CT 06269, USA; National Key Laboratory of Crop Genetics & Germplasm Enhancement and Utilization, Nanjing Agricultural University, Nanjing 210095, `China; College of Horticulture, Nanjing Agricultural University, Nanjing 210095, China

## Abstract

Chromatin structure plays a critical role in the regulation of dynamic gene expression in response to different developmental and environmental cues, but as yet their involvement in fruit ripening is not well understood. Here, we profile seven histone modifications in the woodland strawberry (*Fragaria vesca*) genome and analyze the histone modification signatures during ripening. Collectively, segments painted by the seven marks cover ~85% of the woodland strawberry genome. We report an eight-state chromatin structure model of the woodland strawberry based on the above histone marks, which reveals a diverse chromatin environment closely associated with transcriptional apparatus. Upon this model we build a chromatin-centric annotation to the strawberry genome. Expression of many genes essential for fruit ripening, such as abscisic acid catabolism, anthocyanin accumulation and fruit softening, are associated with shifts of active genic states and polycomb-associated chromatin states. Particularly, the expression levels of ripening-related genes are well correlated with histone acetylation, indicating a regulatory role of histone acetylation in strawberry ripening. Our identification of the chromatin states underpinning genome expression during fruit ripening not only elucidates the coordination of different pathways of morphological and metabolic development but also provides a framework to understand the signals that regulate fruit ripening.

## Introduction

Gene expression in eukaryotes is orchestrated by the interaction of transcription factors and the transcriptional apparatus with chromatin, the basic unit of which is the nucleosome, comprising ~146 bp of DNA wrapped around a histone octamer. Covalent modifications on the N-terminal tails of core histones regulate gene expression by affecting the accessibility of DNA for transcription, histone–DNA interactions, and recruitment of transcription factors or chromosomal proteins. A subset of modifications, such as the methylation at histone 3 lysine 9 (H3K9me) and histone 3 lysine 27 (H3K27me) sites, are associated with gene silencing, while some other modifications, such as histone acetylation and histone 3 lysine 4 trimethylation (H3K4me3), indicate gene transcription [1]. Acetylation disrupts the interaction between histones and DNA and allows an open chromatin environment that promotes transcription [2].

Eukaryotic chromosomes are organized into structurally and functionally distinct domains called heterochromatin and euchromatin, originally identified microscopically as dense- and light-staining chromosomal regions [3]. Generally speaking, euchromatin is gene-rich and histone acetylation elevated, with higher transcriptional activity, whereas heterochromatin is much less permissive for gene transcription [4]. Two forms of heterochromatin are present in eukaryotic cells, the H3K9me2-enriched constitutive heterochromatin and the H3K27me3-enriched polycomb group (PcG)-associated facultative heterochromatin. Constitutive heterochromatin, mainly formed in the gene-poor pericentromeric regions, is important for transposable element (TE) silencing and the maintenance of chromosome stability [4]. In contrast, facultative heterochromatin resides sparsely along the chromosomes, and plays essential roles in regulating the genes involved in growth and development [5].

Previous studies in the genome-wide profiling of histone modifications in plants, e.g. *Arabidopsis* [6–8], rice [9], maize [10], and *Brassica napus* [11], have contributed substantially to our understanding of the molecular mechanisms and potential functions of chromatin composition. In *Arabidopsis*, H3K4me2, H3K4me3, H3K27me3, and H3K56ac are mostly found in euchromatin; H4K20me1 and H3K9me2 are found in heterochromatin [6]. High levels of H3K4me2, H3K4me3, H3 acetylation, H3K36me3, and H2Bub are associated with transcribed regions [7]. Work on dynamic chromatin state profiling in shoot regeneration also reveals that the transcription regions are captured by states associated with high levels of H3K36me3, H3K4me1, and RNAP II, and, similar to animals, H3K9me2 and H3K27me3 are related to inactive genes [8]. In rice, a high level of H3K4me1 is associated with transcription and the strong transcription chromatin state is marked with RNAP II occupancy and low enrichment of retrotransposons and DNA transposons [9]. Further, changes in chromatin states are associated with various biological events. In shoot regeneration in *Arabidopsis*, genes embedded in the PcG-associated heterochromatin states are related to leaf morphogenesis and meristem determinacy [8]. In wheat, it is found that some H3K27ac-related genes are enriched within quantitative trait loci regions that govern nitrogen-use efficiency [12]. Nevertheless, our understanding of the underlying mechanism of fruit ripening and its implications for evolution and agricultural selection remains limited by the unknown chromatin organization in fruits.

Fruit ripening is a highly coordinated process influenced by both hormonal and environmental factors. Ethylene initiates the ripening and senescence of climacteric fruits such as tomato and apple. By contrast, abscisic acid (ABA) triggers the ripening process of non-climacteric fruits such as strawberry and grape, which typically proceeds only while the fruit is on the plant [13, 14]. Recent studies suggest a tight link between epigenetic regulation and plant hormone signaling [13, 15, 16]. Further, histone modifications are suggested to be coordinated with the ripening of climacteric fruits. Biochemical data and transient expression experiments indicate that histone deacetylases (HDACs) regulate ethylene biosynthesis during the ripening of apple [17] and banana [18]. The Polycomb Repressive Complex 1 (PRC1)-like protein, SlLHP1b (Like Heterochromatin Protein 1b), represses tomato fruit ripening via colocalization with H3K27me3 [19]. FruitENCODE data also demonstrate a close association between H3K27me3 levels and expression levels of ripening-related genes in dry and fleshy fruits [20]. Recent studies suggest a regulatory role of RNA-directed DNA methylation [21] and N(6)-methyladenosine RNA modification [22] in the strawberry ripening process as well. However, little is known about whether and how histone modifications are involved in the ripening of non-climacteric fruits such as strawberry.

In this study, we profiled histone modifications in immature and mature fruits of the woodland strawberry, *Fragaria vesca*. This work generates a comprehensive epigenomic annotation to the strawberry genome and provides novel insights into how the ripening of non-climacteric fruits might be regulated by chromatin composition remodeling.

## Results

### Characterization of histone modifications along genic sequences in strawberry fruits

**Figure 1:**
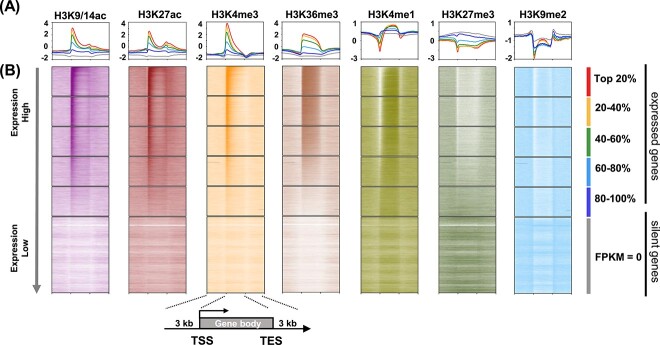
Distribution patterns of the seven histone modifications along genic sequences. **A** Metagene analysis of seven histone marks (H3K9/K14ac, H3K27ac, H3K4me3, H3K36me3, H3K4me1, H3K27me3, and H3K9me2) along the gene body. The expressed genes (FPKM >0) were evenly classified into five groups (top 20%, 20–40%, 40–60%, 60–80%, 80–100%). **B** Enrichment heat maps showing the location and extent of the seven histone modifications relative to boundaries of expressed or silent genes. The gene body (from transcription start site [TSS] to transcription end site [TES]), 3-kb sequences upstream of the TSS, and 3-kb sequences downstream of the transcriptional end site are profiled. Color intensity indicates the relative enrichment of a given mark along genic sequences (normalized independently for each mark). Data are from red-stage fruits of *F. vesca.*

To characterize the enrichment patterns of histone modifications in strawberry, we performed chromatin immunoprecipitation coupled with high-throughput sequencing (ChIP-seq) for seven histone marks in immature and mature fruits of the woodland strawberry *Fragaria vesca* (‘Ruegen’) (Fig. 1). In both immature pre-T-stage fruits (pre-turning stage, when fruit has white flesh with red achenes) and mature red-stage fruits, enrichment levels of three active marks, H3K9/K14ac, H3K27ac, and H3K4me3, were highly correlated with one another (Supplementary Data Fig. S1A). Enrichment levels of the marks indicative of transcription elongation, H3K4me1 and H3K36me3, were moderately correlated with each other (Supplementary Data Fig. S1A). In contrast, correlation of enrichment peaks of the two silent marks, H3K27me3 and H3K9me2, representing the PcG-associated facultative heterochromatin and repeat-rich constitutive heterochromatin, respectively, was weak (Supplementary Data Fig. S1A).

Gene expression is related to the composition and level of histone modifications. Then, we further divided the expressed genes (FPKM >0, red-stage fruit) into five groups according to the expression levels with about the same number of genes (Supplementary Data Table S1). Combined with ChIP-seq data, the metagene distribution of histone modifications for each group was plotted (Fig. 1A). In red fruits, the active marks H3K9/K14ac, H3K27ac, and H3K4me3 were enriched in the body of actively transcribed genes and reached the highest level downstream of the transcription start site (TSS; Fig. 1A and B). The higher the expression level of the group, the higher the enrichment level of these marks along the gene body. On the other hand, enrichment levels of the three active marks were relatively low throughout the silent gene loci. The results indicate a positive correlation between gene expression level and enrichment of these three marks, as expected.

The transcription elongation mark H3K36me3 remained at a high level throughout the gene body of expressed genes (Fig. 1). Similar to the above-mentioned three active marks, the H3K36me3 level was relatively low in silent genes and positively correlated with gene expression. Another transcription elongation mark, H3K4me1, was enriched along the gene body of expressed genes while depleted in silent genes (Fig. 1). It was noted that, of the five groups of expressed genes, the one with highest levels of expression had relatively higher H3K4me1 levels in the gene body, while around the transcription start site (TSS) the H3K4me1 level was negatively instead of positively correlated with expression level. Thus, there is no simple positive correlation between H3K4me1 and expression. On the contrary, the levels of silent marks H3K27me3 and H3K9me2 were depleted along active genes. Gene expression was negatively correlated with the enrichment level of H3K27me3 both around the TSS and along the gene body of expressed genes, and was negatively correlated with the enrichment level of H3K9me2 around the TSS (Fig. 1).

### Eight chromatin states based on the enrichment of histone modifications are closely associated with transcriptional status in strawberry fruits

**Figure 2:**
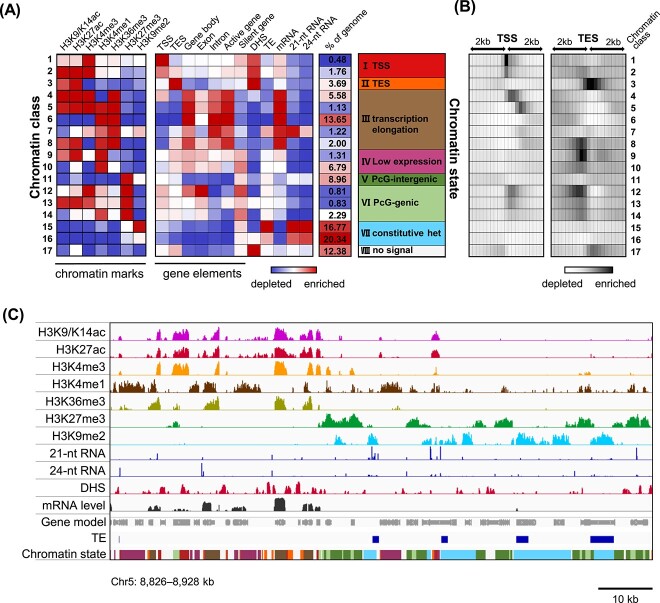
Eight chromatin states based on the distribution of histone modifications are closely associated with transcriptional status in the woodland strawberry. **A** Eight chromatin states identified in the red-stage strawberry fruits by the ChromHMM method. Each chromatin state is defined by a combinatorial pattern of enrichment or depletion for the profiled seven histone modifications. The eight chromatin states are closely associated with transcriptional status, and characterized by TSS-proximal sequences (state I, classes 1 and 2), TES-proximal sequences (state II, class 3), transcription elongation (state III, classes 4–8), low expression (state IV, classes 9 and 10), intergenic or genic sequences in H3K27me3-enriched PcG-associated regions (state V, class 11; state VI, classes 12–14), H3K9me2-enriched constitutive heterochromatin (state VII, classes 15 and 16), and sequences with no obvious histone marks (state VIII, class 17), respectively. The second panel shows genomic and transcriptional features for each state. **B** Distribution of each chromatin class along the gene body. Each box represents 200 bp. **C** Browser view showing distribution patterns of the profiled seven histone modifications, small RNAs, DHSs, and mRNAs associated with gene loci. Colors for the eight chromatin states are indicated in panel **A**. Data are from red-stage fruits of *F. vesca.*

To characterize the chromatin structure of the *F. vesca* genome in fruits, we ran ChromHMM [23, 24] to profile the combinatorial presence or absence of the seven histone marks in fruits along each chromosome (Fig. 2, Supplementary Data Figs S1B and C and S2). We set the class number as 13–30 for chromatin class learning by ChromHMM. The result of the 17-class model from the learning process was picked based on output parameters. Then, the 17 chromatin classes were further merged into eight regional and transcription-related states based on both enrichment of histone marks and genomic occurrences (e.g. annotated genic or *cis*-regulatory regions). To explore the features of the identified chromatin states, we further examined the enrichment of various gene elements, TEs, and DHSs (DNaseI hypersensitive sites) [20], as well as gene transcripts and small RNA data, which showed distinct characteristics of each chromatin state. States I–III, state IV, states V–VI, and state VII were associated with active genic regions, low-expression regions, H3K27me3-enriched facultative heterochromatin, and H3K9me2-enriched constitutive heterochromatin, respectively (Fig. 2A–C, Supplementary Data Fig. S1B and C). State VII was barely enriched for the seven histone marks. Overall, segments painted by one or more of the profiled histone marks covered 86.1 and 87.6% of the genome in immature pre-T-stage fruits and mature red-stage fruits, respectively (Fig. 2A, Supplementary Data Fig. S1B).

We also used ChromHMM to calculate the transition parameter, which illustrated the proximal genomic locations among each chromatin class [23, 24]. The transition probability from each class to each other class automatically captured the positional bias of the 17 classes, such as broad transcribed domains characterized by classes 4–8, PcG domains grouped by classes 11–14, and constitutive silent domains captured by classes 15–16 (Supplementary Data Fig. S3). The result of transition parameter analysis supports our eight-state model.

Of particular interest are states I–IV associated with transcriptionally active or silent genes (Fig. 2A and B). TSS-proximal sequences were identified by state I, marked by prominent enrichment levels of H3K9/K14ac, H3K27ac, and H3K4me3, and a high density of DHSs indicative of open chromatin amenable for transcription. Sequences surrounding the transcription end site (TES) were identified by state II, marked by high levels of H3K27ac and DHSs, and moderate levels of H3K9/K14ac. The signature of transcription elongation associated with H3K36me3 deposition was captured by state III. In state III, class 4 enriched for TSS-proximal features (H3K9/K14ac, H3K27ac, and H3K4me3) and the elongation mark H3K36me3, was more frequently located in the genic regions close to the TSS. This is consistent with the moderate transition probability from state I to class 4 (Supplementary Data Fig. S3), while classes 5 and 6, enriched for the elongation marks H3K36me3 and H3K4me1, were preferentially residing in the middle part of active genes. Class 7 was distinguished as another set of genic sequences containing medium levels of elongation marks as well as enrichment of H3K9me2, representing TE-rich introns embedded in transcribed genes. Lastly, a chromatin state associated with the body of silent or low-expressed genes was captured by state IV, distinguished by enrichment of H3K4me1 but lack of H3K36me3. In state IV, class 9 was also enriched for H3K9/K14ac and H3K27ac while class 10 was not.

Genomic sequences associated with condensed heterochromatin were captured by the eight chromatin states as well. PcG-associated facultative heterochromatic regions were captured by states V and VI, with prominent levels of H3K27me3 (Fig. 2). Specifically, state V and state VI were characterized by PcG-associated intergenic and genic sequences, respectively. The PcG-genic state VI consisted of three classes. Classes 12 and 14 were enriched for H3K4me3 and H3K4me1, representing chromatin features of the front and back parts of the PcG-associated gene body, while class 13 was enriched for the active marks H3K9/K14ac, H3K27ac, and H3K4me3, representing features of PcG-associated expressed sequences (Fig. 2A and B). The H3K9me2-enriched constitutive heterochromatin was identified by state VII, obtaining high levels of TEs and small RNAs (Fig. 2A). Additionally, the transition probability of state VII to other states was low, indicating that state VII is distinct from others (Supplementary Data Fig. S3). Overall, the eight chromatin states we have identified in strawberry fruits reveal histone modification patterns closely associated with various gene elements and the transcription status of the underlying sequences.

### A chromatin-centric annotation to the strawberry genome

**Figure 3:**
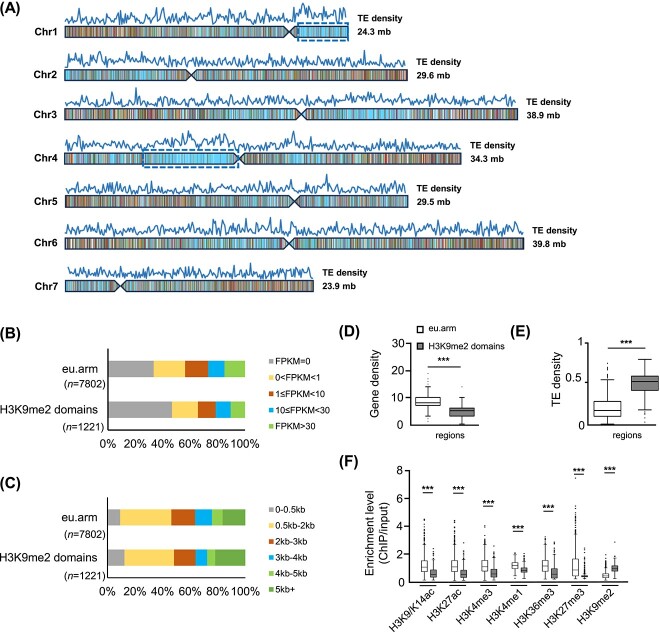
A chromatin-centric annotation to the woodland strawberry genome created by the eight chromatin states reveals its chromatin organization. **A** Genome-wide karyotype view of the chromatin domains defined by the eight chromatin states (as described and colored in Fig. 2A) identifies large blocks of H3K9me2-enriched constitutive heterochromatin. TE density is illustrated above each chromosome. Chromosomes 1 and 4 have broad H3K9me2-enriched domains (blue-framed) indicative of pericentric heterochromatic regions (chr1, 19 600 000–24 346 798; chr4, 6 650 000–15 000 000). **B** Bar graph illustrating expression levels of genes embedded in the H3K9me2-enriched constitutive heterochromatin domains (H3K9me2 domain) and euchromatic chromosome arms (eu.arm) on chromosomes 1 and 4. **C** Distribution of gene length in H3K9me2-enriched constitutive heterochromatin domains and eu.arm regions on chromosomes 1 and 4. **D**–**F** Gene density (**D**), TE density (**E**), and enrichment levels of the profiled seven histone modifications (**F**) in the H3K9me2-enriched constitutive heterochromatin domains and eu.arm on chromosomes 1 and 4. The H3K9me2-enriched constitutive heterochromatin domains and eu.arm regions were partitioned into 50-kb intervals, and the TE density and enrichment levels of each mark were calculated accordingly. Boxplots in **D**–**F** include a median with quartiles and outliers above/below the top/bottom whiskers. *n* = 914 for eu.arm, *n* = 261 for H3K9me2-enriched domains. ^*^^*^^*^ indicates significantly different means (*P <* 10^−10^, Student’s *t*-test). Data are from red-stage fruits of *F. vesca.*

The eight chromatin states associate each genomic location with a particular state, generating a chromatin-centric annotation to the strawberry (*F. vesca*) genome. The genome-wide karyotype view of the chromatin domains defined by the eight states revealed several features of large-scale organization. We observed broad H3K9me2-enriched domains (with relatively high-density TEs and large blocks of H3K9me2 peaks) on chromosomes 1 and 4 near centromeres, indicating pericentric heterochromatic regions (Fig. 3A, Supplementary Data Fig. S4).

To explore the genome features of the distinct regions, we analyzed gene expression in the broad H3K9me2-enriched domains on chromosomes 1 and 4, and the remaining regions along euchromatic chromosome arms (eu.arm) on the same chromosomes. The result revealed that, compared with the eu.arm regions, the H3K9me2-enriched domains had a higher proportion of silent genes as well as a lower proportion of highly expressed genes (Fig. 3B). The gene length distribution analysis showed that the H3K9me2-enriched domains contained a higher proportion of both short genes (<500 bp) and long genes (>5000 bp) than eu.arm regions (Fig. 3C). Also, the gene density in the eu.arm regions was almost twice as that in the H3K9me2-enriched domains (8.43 vs 4.67/50 kb; Fig. 3D). Additionally, these H3K9me2-enriched domains had prominent TE density (Fig. 3E) and relatively lower levels of active marks, and were less painted with H3K27me3 (Fig. 3F). Thus, the eight chromatin-state model highlights large blocks with prominent H3K9me2 enrichment indicative of pericentric heterochromatin, underscoring the importance of the chromatin landscape relating genome organization to the regulation of gene expression.

### Correlation between expression levels of ripening-related genes and switches of corresponding local chromatin states during strawberry fruit ripening

Strawberry fruit ripening is coupled with transcriptional reprogramming of key genes. We profiled the chromatin environment of previously demonstrated ripening-related genes to study whether their dynamic expression was associated with changes of chromatin structure. The ripening-related genes we looked into included those involved in ABA homeostasis [25, 26], anthocyanin biosynthesis and transport [27–38], cell wall metabolism [39–42], sugar biosynthesis [43, 44] and aroma formation [45]. Twenty-five of the investigated genes were differentially expressed between pre-T-stage and red-stage fruits, with 23 upregulated and 2 downregulated (Supplementary Data Table S2). One gene (*Fv4CL*) was embedded in the large blocks of putative pericentric constitutive heterochromatin. Of the other 24 genes, 14 were located in H3K27me3-enriched segments while other 10 were in H3K27me3-less regions (Supplementary Data Table S2).

**Figure 4:**
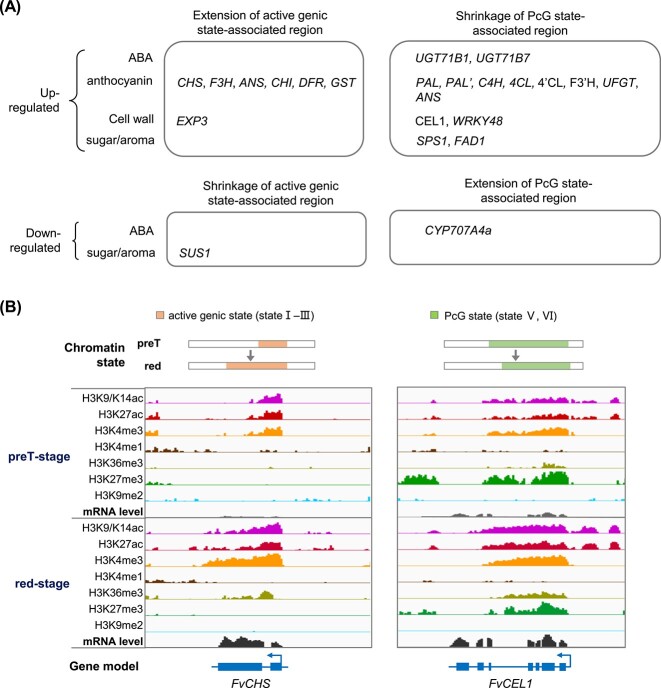
Close association between expression levels of ripening-related genes and switches of their local chromatin states during strawberry fruit ripening. **A** Switches of chromatin states of ripening-related genes are associated with expression changes during ripening. Previously identified ripening-related genes were analyzed, and 21 DEGs (pre-T stage vs red stage) with switches of chromatin states were profiled. Italic type indicates genes whose upregulation or downregulation is associated with differential H3K9/K14ac peaks and positively correlated with H3K9/K14ac enrichment along gene loci. The gene body, 500 bp upstream of the TSS, and 200 bp downstream of the TES were considered. **B** The chalcone synthase gene *FvCHS* is upregulated during ripening, associated with an extension of regions of active genic states (states I–III of the eight chromatin states). The endo-1,4-β-glucanase gene *FvCEL1* is upregulated, associated with a shrinkage of PcG-associated states (states V and VI of the eight chromatin states). Refer to Fig. 2A for characterization of the eight chromatin states.

Although the 25 ripening-related genes were in different chromatin environments, expression changes in 23 of these genes were correlated with switches in chromatin states along gene loci (Fig. 4A). The regions considered included the gene body, 500 bp upstream of the TSS and 200 bp downstream of the TES. Seven upregulated genes and one downregulated gene were associated with an extension or shrinkage of active genic states (states I–III) but not PcG-associated states (states V and VI), e.g. *FvCHS* for anthocyanin accumulation (Fig. 4A and B). Fourteen of the upregulated genes and one downregulated gene were associated with switches of H3K27me3-enriched PcG-associated states (states V and VI), e.g. *FvCEL1* and *FvWRKY48* for cell wall metabolism, *FvFAD1* for aroma production, coupled with significant loss of H3K27me3 (Fig. 4A and B). It was noted that some genes were associated with switches of both the active genic state and PcG states (e.g. *FvPAL*, *Fv4CL*), indicating cross-talk of chromatin states. Collectively, our data demonstrate a close association between gene expression levels of ripening-related genes and their local chromatin states, suggesting that histone modifications are involved in fruit ripening.

### Histone acetylation positively correlates with transcriptional reprograming of ripening-related genes

**Figure 5:**
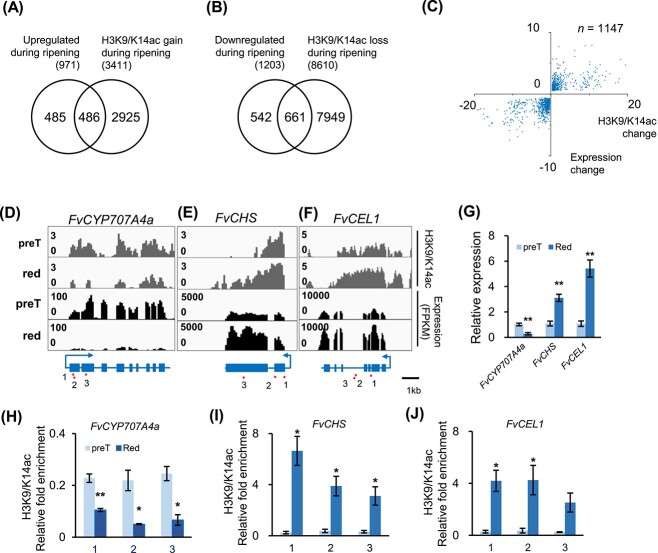
Positive correlation between histone acetylation levels and expression levels of ripening-related genes. **A**, **B** Venn diagrams illustrate that 50.1% (486/971) of upregulated genes and 54.9% (661/1203) of downregulated genes are associated with differentially acetylated peaks (H3K9/K14ac) during ripening (pre-T stage vs red stage). The gene body, 500 bp upstream of the TSS and 200 bp downstream of the TES were considered. **C** Positive correlation between H3K9/K14ac changes and gene expression changes during ripening. We profiled 1147 DEGs associated with differential H3K9/K14ac peaks. H3K9/K14ac and expression changes are displayed as log_2_(FC). **D–J** Positive correlation between expression levels and H3K9/K14ac levels of an ABA oxidative degradation gene *FvCYP707A4a* (**D**, **G**, **H**), a chalcone synthase gene *FvCHS* (**E**, **G**, **I**), and an endo-1,4-β-glucanase gene *FvCEL1* (**F**, **G**, **J**). RT–qPCR data in **G** illustrate expression levels relative to *FvCHC1*. ChIP–qPCR data in **H**–**J** illustrate H3K9/K14ac levels relative to *FvEF1α.* Red lines in **D**–**F** indicate the DNA fragments profiled in ChIP–qPCR experiments shown in **H**–**J**. Error bars represent mean ± s.e.m. of *n* = 3 experiments. ^*^ and ^*^^*^ denote statistical significance, *P* < 0.05 and 0.01, respectively; Student’s *t*-test. Differential H3K9/K14ac peaks (|log_2_FC| > 0.3, FDR < 0.05); DEGs (FC >1.5, FDR < 0.05).

Our chromatin state analysis reveals that transcriptional reprogramming of ripening-related genes is correlated with switches of the H3K9/K14ac, H3K27ac, and H3K4me3-enriched active states and H3K27me3-enriched silent states. Thus, we further analyzed the correlation between gene expression and differential enrichment peaks of histone marks. From the pre-T-stage to red-stage fruits, there are 2174 differentially expressed genes (DEGs), including 971 upregulated and 1203 downregulated ones (pre-T-stage vs red-stage, FDR [false discovery rate] <0.05, FC [fold change] >1.5). Co-analysis of DEGs and differential acetylation/methylation peaks (FDR < 0.05, |log_2_FC| > 0.3) revealed that, of the upregulated genes, 50.1, 19.3, 22.4 and 3.8% were associated with gain of H3K9/K14ac, H3K27ac, H3K4me3, and H3K36me3, respectively, while 10.9% were associated with loss of H3K27me3 (Fig. 5A and B, Supplementary Data Figs S5 and S6). Of the downregulated 1203 genes, 54.9, 37.2, 47.4 and 4.82%were associated with loss of H3K9/K14ac, H3K27ac, H3K4me3, and H3K36me3, respectively, and 8.1% were associated with gain of H3K27me3 (Fig. 5A and B, Supplementary Data Figs S5 and S6). Our results indicate that the reprogramming of gene expression during ripening is well correlated with histone acetylation, indicating an important role of histone acetylation in strawberry ripening.

Histone acetylation represents an open chromatin environment accessible for transcription apparatus. Consistently, the above analysis of DEGs suggests a close association between gene expression and H3K9/K14ac levels. Gene ontology enrichment analysis showed that these differential H3K9/K14ac peak-associated DEGs were enriched in various pathways, such as photosynthesis, lignan biosynthesis, and chlorophyll biosynthesis (Supplementary Data Fig. S7, Supplementary Data Tables S3 and S4). Moreover, expression levels of 22 of the 25 ripening-related DEGs were associated with differential acetylation peaks and positively correlated with H3K9/K14ac levels (Supplementary Data Table S2). Both transcriptome and RT–qPCR data revealed that the expression level of the ABA catabolic gene *FvCYP707A4a* was downregulated 71.9%, which corresponded to a significant reduction in H3K9/K14ac levels (Fig. 5D, G and H, Supplementary Data Fig. S8A). Similarly, the expression of the anthocyanin biosynthesis gene *FvCHS* was upregulated by >2-fold, a change that was associated with a significant increase in H3K9/K14ac levels along the gene locus (Fig. 5E, G and I, Supplementary Data Fig. S8B). Additionally, the expression of *FvCEL1*, a gene involved in cell wall metabolism, was upregulated by >2-fold, paralleling a >2-fold increase in H3K9/K14ac enrichment (Fig. 5F, G and J, Supplementary Data Fig. S8C). These results demonstrate that local H3K9/K14ac enrichment levels are positively correlated with expression levels of ripening-related genes, indicating histone acetylation as an epigenetic mark essential for strawberry fruit ripening.

## Discussion

In this study, we profile the distribution of seven histone modifications and characterize eight chromatin states in woodland strawberry fruits based on these histone marks. Transcriptional reprogramming of many genes essential for strawberry fruit ripening is associated with shifts of local active genic chromatin states and PcG-associated states, and positively correlated with histone acetylation. Our chromatin-centric annotation at both chromosome-wide and element-specific scales during fruit ripening is highly valuable for understanding of the organization of the strawberry genome and the molecular mechanisms of fruit epigenome regulation.

The distribution patterns of histone modifications along genic sequences in *F. vesca* fruits are characterized in our study. The active marks H3K9/K14ac, H3K27ac, H3K4me3, and H3K36me3 are enriched in the gene body and positively correlated with gene expression, and are consistent with previous studies such as those in *Arabidopsis* and maize [8, 46]. The repressive mark H3K27me3 is depleted in the gene body of active genes and negatively correlated with gene expression, as expected. On the other hand, the elongation mark H3K4me1 is roughly positively correlated with expression in the gene body while negatively correlated with expression around the TSS in strawberry. These results indicate that the local chromatin environment with complex components interlinking various factors influences expression activity. H3K9me2 serves as a mark for constitutive heterochromatin in both plants and animals [47]. Our chromatin-centric annotation reveals large blocks of H3K9me2-enriched domains, which have high TE density, high levels of small RNAs, and relatively low levels of active marks and transcription. Some blocks of H3K9me2-enriched domains are near to the predicted centromeres in the woodland strawberry [48], indicative of pericentric heterochromatin. Our eight-state chromatin structure model based on histone modifications gives a new insight into the chromatin landscape in strawberry fruits.

The identified eight chromatin states are closely associated with transcriptional activity. State I represents TSS-proximal sequences with prominent enrichment levels of H3K9/K14ac, H3K27ac, and H3K4me3, and a high density of DHSs, similar to previously reported active TSS regions in *Arabidopsis*, wheat, rice, and *B. napus* [8, 9, 11, 12]. In wheat, for example, the transcription initiation state has high levels of H3K27ac, H3K4me3, and H3K36me3; the elongation state is characterized by high levels of H3K36me3, moderate levels of H3K27ac and H3K4me3; and another TE-rich transcription elongation state has high levels of H3K36me3, and weak H3K27ac, H3K4me3, and H3K9me3 [12]. We have identified similar classes in state III in our model as well. These results suggest the presence of consistent chromatin signatures associated with transcriptional elements in plants.

Epigenetic modifications exhibit specificity across tissues as well as species, which contributes to distinct agronomic traits [11, 12, 47]. In rice, a distinct chromatin state enriched for both the active mark H3K4me3 and the silent mark H3K27me3 is associated with DEGs associated with the salt stress response in seedlings and roots [47]. In *B. napus* a novel bivalent chromatin state enriched for the elongation mark H3K4me1 and the silent mark H3K27me3 is associated with tissue-specific gene expression [11]. Similar chromatin classes (classes 12–14) have been identified in our chromatin state model as well. These results indicate that genes underlying these bivalent chromatin classes may have specific functions during strawberry fruit ripening.

An increasing number of studies suggest a close association between epigenetic regulation and plant hormone-mediated fruit ripening. It has been reported that changes in chromatin structure are associated with climacteric fruit ripening through regulating the ethylene pathway [17, 18]. Experimental evidence suggests that DNA methylation [21] and mRNA m^6^A modification [22] are involved in strawberry fruit ripening. Recently, histone acetylation has been revealed to be associated with anthocyanin accumulation in grapes [38]. In this study we show that strawberry ripening-related genes display histone mark-based chromatin structure changes during ripening. An extension/shrinkage of regions with active genic states or PcG-associated states has been observed along key genes involved in ABA homeostasis, accumulation of anthocyanin, and alterations in fruit texture, as well as sugar and aroma. Thus, chromatin state changes may play a key role in transcriptional reprogramming in strawberry fruit ripening.

Epigenomic modifications mediate between genes and environment in growth and developmental processes. Our work provides a histone modification-based chromatin annotation to fruits of a non-climacteric plant species. Our chromatin profiling for *F. vesca* genes reveals that the chromatin organization for active transcription is closely associated with the local chromatin packaging. We conclude that patterns of euchromatin and heterochromatin packaging show great complexity and plasticity in strawberry, which may provide a chromatin landscape basis for breeding and mechanistic studies.

## Materials and methods

### Plant materials and growth conditions

The woodland strawberry (‘Ruegen’) plants were grown in a greenhouse (16 h light/8 h dark at 23 ± 3°C) for collection of fruit materials. Strawberry fruits at the pre-turning stage (pre-T: white flesh with red achenes) or the red stage were harvested and deseeded for the following ChIP and small RNA-seq experiments.

### ChIP-seq experiments and data processing

ChIP-seq experiments and data processing were performed as previously described [49]. Two biological replicates were performed for each ChIP experiment. In brief, 5 g of fruits (without seeds) were ground to powder in liquid nitrogen, suspended in extraction buffer 1, and homogenized on ice. Homogenates were centrifuged at 4500 g for 10 min at 4°C. The pellet was resuspended in extraction buffer 2 and centrifuged at 4500 g for 10 min at 4°C. The pellet was resuspended in micrococcal nuclease buffer for chromatin shearing. The nuclei were digested into fragments of 150–500 bp by micrococcal nucleases (4 U/μl, M0247S, NEB) for 10 min at 37°C. Then, 100 μl of chromatin was set as the input, and the remaining chromatin was aliquoted for ChIP experiments. Each 500 μl of chromatin was pre-cleaned with Dynabeads^®^ Protein A (Thermo Fisher, catalog no. 10001D), incubated with the proper antibody and pulled down by Dynabeads^®^ Protein A. DNA was extracted and diluted using the RaPure Plant DNA Mini Kit (Magen, D3187–03). Library amplification and sequencing were performed by BGI on the MGISEQ-2000 platform. The antibodies used were anti-acetyl-histone H3 (Lys 9/Lys 14) (Diagenode, C15410200-20), anti-acetyl-histone H3 (Lys 27) (Abcam, ab4729), anti-trimethyl-histone H3 (Lys 4) (Abcam, ab8580), anti-monomethyl-histone H3 (Lys 4) (Abcam, ab8895), anti-trimethyl-histone H3 (Lys 36) (Abcam, ab9050), anti-trimethyl-histone H3 (Lys 27) (Abcam, 6002), and anti-dimethyl-histone H3 (Lys 9) (Abcam, ab1220). The specificity of the antibodies has been validated by western blot, ChIP-chip or ChIP-array assays [50].

Clean data were aligned to the *F. vesca* genome (v6.0) [48] by Bowtie2 [51]. Sorted aligned files were used for peak calling and narrow peak files were obtained using MACS2 with default model parameters. Consensus peaks from the two replicates (with >60% overlap) were used for differential peak analyses by using DiffBind (v3.6.1). Reads were counted based on consensus peaks and normalized by library size using DBA.NORM.LIB with the default setting. Differential peaks were filtered with FDR < 0.05, |log_2_FC| > 0.3. For each gene, if regions within the gene body, 500 bp upstream of the TSS, and 200 bp downstream of the TES overlap with differential H3K9/K14ac peaks, the gene is considered to exhibit acetylation changes. The ChIP-seq data produced in this study are listed in Supplementary Data Table S5.

### Chromatin state analysis

The workflow for building the eight chromatin states is illustrated in Supplementary Data Fig. S2. ChromHMM [23, 24] was applied to learn the chromatin states, annotate their occurrences across the genome, and compute the enrichments. Both biological replicates for each ChIP were used for building the model. Model learning and segmentation were performed with varying numbers of classes (13–30 classes) with default parameters. Then, the 17-class model was selected and subsequently merged into eight states for their biological relevance (for instance modifications localized to specific functional regions of genes, or associated with constitutive or regulated silencing, or correlated with ongoing transcriptional elongation). The enrichment of genomic features was calculated by OverlapEnrichment with default parameters. The distribution of each chromatin class along the gene body (as in Fig. 2B and Supplementary Data Fig. S1C) was calculated by NeighborhoodEnrichment with default parameters. The emission and enrichment files were used for drawing heat maps in Excel.

### Transcriptome data processing

The transcriptome data for pre-T-stage and red-stage fruits produced by previous studies were used [52]. The reference genomes used were *F. vesca* v6.0 (https://www.rosaceae.org/). The clean data were mapped to the corresponding reference genome by HISAT2 [53]. The mapped reads were sorted for the calculation of reads based on the *F. vesca* genome annotation (v6.0). Differential expression analysis was performed by the edgeR (1.18.0) package with FDR. The DEGs were filtered with fold change >1.5 and FDR <0.05.

### Small RNA sequencing and data processing

The total RNA was extracted by the CTAB-pBIOZOL reagent, qualified and quantified using a Nano Drop (NanoDrop, Madison, USA) and Agilent 2100 bioanalyzer (Agilent, Santa Clara, USA). One microgram of total RNA for each sample was used for library preparation and sequencing was performed by BGI on the MGISEQ-2000 platform. The clean data were aligned to the *F. vesca* v6.0 genome using Bowtie [54]. After indexing and sorting with default settings, the 21-nt and 24-nt small RNAs were predicted and filtered by sRNAminer [55].

### Quantitative real-time PCR assays

Total RNA was extracted with the E.Z.N.A.^®^ RNA Kit. The cDNA was synthesized using HiScript II Q RT SuperMix for qPCR (Vazyme). RT–qPCR was run on the CFX96 Touch Real-Time PCR Detection System (Bio-Rad) using ChamQ SYBR qPCR Master Mix (Vazyme). Three biological replicates and three technical replicates were performed. *FvCHC1* was used as the internal control, and the relative expression levels were normalized using the 2^−∆∆CT^ method [56]. The RT–qPCR primers are listed in Supplementary Data Table S6.

ChIP–qPCR experiments were performed to measure H3K9/K14ac levels of the genes of interest. Three biological repeats and three technical replicates were performed for each experiment. *FvEF1α* was used as the internal control. All ChIP–qPCR primers are listed in Supplementary Data Table S6.

## Supplementary Material

Web_Material_uhae158

## Data Availability

The sequencing data used in this study, the corresponding processed data files for visualization, and the genomic occurrence of the eight chromatin states are available from Gene Expression Omnibus under GSE208640 (https://www.ncbi.nlm.nih.gov/geo/query/acc.cgi?acc=GSE208640).
