# Supplementary material for: Dynamic histone modification signatures coordinate developmental programs in strawberry fruit ripening
Source: Hortic Res. 2024 Jun 7;11(8):uhae158. doi: 10.1093/hr/uhae158 (PMC11298626; doi:10.1093/hr/uhae158)
Supplement: Web_Material_uhae158 [file web_material_uhae158.zip › s.figures 202405011HR-revised.pdf]

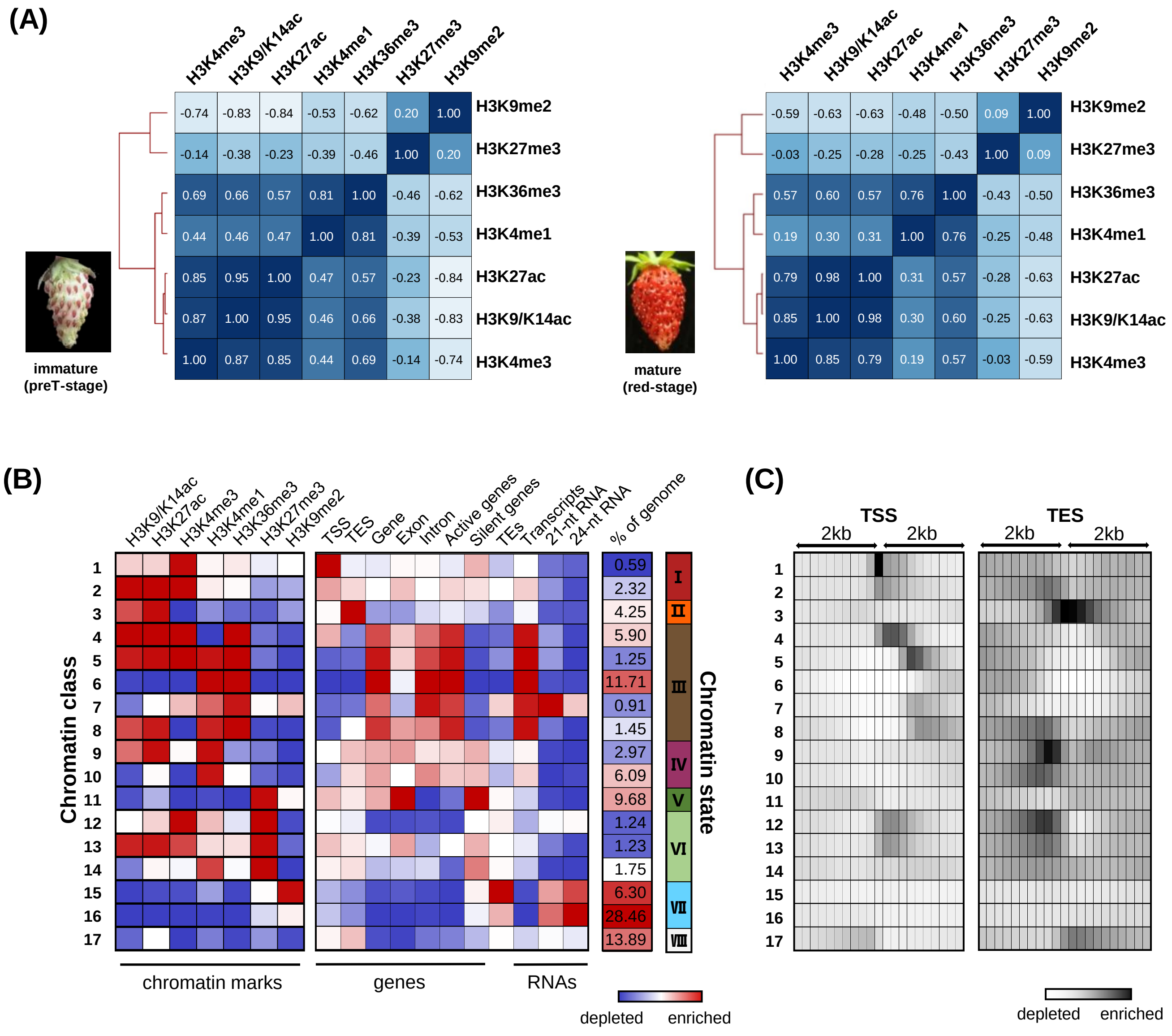

Fig. S1 Histone modifications and chromatin structure of the preT-stage fruits of *F. vesca*. (A) Pearson correlation coefficient of enrichment levels of the seven histone marks in *F. vesca* immature (preT-stage) fruits and mature (red-stage) fruits. **(B)** Eight chromatin states in the preT-stage fruits. Each chromatin state is defined by a combinatorial pattern of enrichment (red) or depletion (blue) for the profiled seven histone modifications. The eight chromatin states are characterized by TSS-proximal sequences (state Ⅰ), TES-proximal sequences (state Ⅱ), transcription elongation (state Ⅲ), low expression (state Ⅳ), intergenic or genic sequences in H3K27me3-enriched PcG-associated regions (state Ⅴ and Ⅵ), H3K9me2-enriched constitutive heterochromatin (state Ⅶ) and sequences with no obvious histone marks (state Ⅷ), respectively. The second panel shows genomic and transcriptional features for each state. **(C)** The corresponding distribution of each chromatin class along the gene body. preT, pre-turning; TSS, transcription start site; TES, transcription end site; PcG, polycomb group. Data from *F. vesca.*

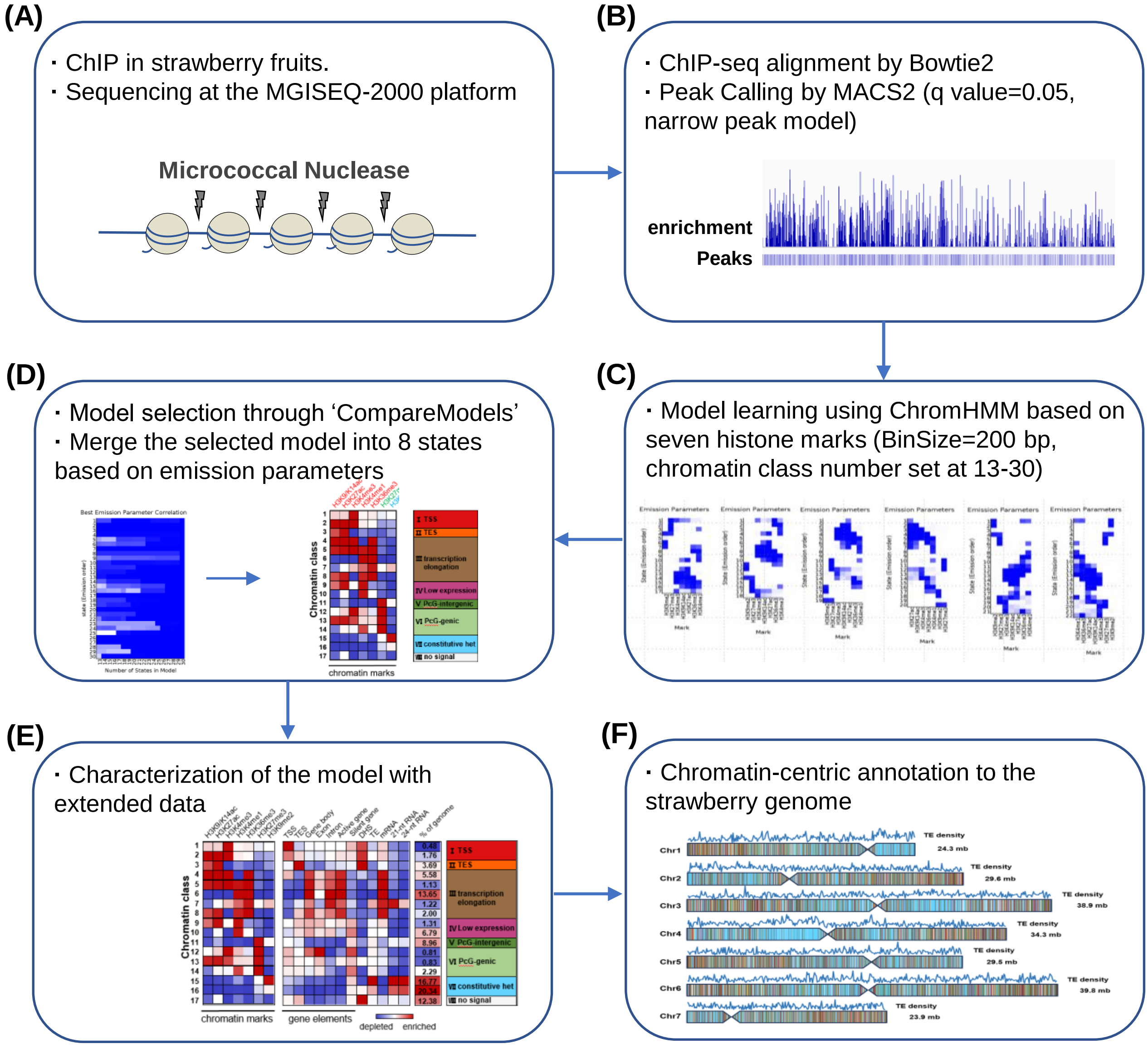

Fig. S2 A workflow for building the eight chromatin states of strawberry by chromHMM. (A) ChIP-seq experiments were performed for seven histone marks (H3K9/K14ac, H3K27ac, H3K4me3, H3K4me1, H3K36me3, H3K27me3 and H3K9me2) in immature and mature fruits. Sequencing data were produced at the MGISEQ-2000 platform at BGI. **(B)** Clean data were aligned to the *F. vesca* genome (v6.0). Narrow peaks were called by MACS2 with q value = 0.05. **(C)** Class number was set from 13 to 30 for chromatin class learning using ChromHMM with 'LearnModel' (convergedelta = 0.001, binsize = 200 bp), which produced 18 original models. ChIP-seq profiles in immature and mature fruits were used as the input. **(D)** 'CompareModels' was applied to show the maximum correlation of each class in the 18 original models. The 17-class model was picked and further merged into eight chromatin states based on emission/output parameters, which represented the enrichment level of each histone mark. **(E)** Characterization of the eight chromatin states with extended data such as gene elements, TEs, as well as gene transcripts and small RNA in immature and mature fruits. **(F)** Building a chromatin-centric annotation to the strawberry genome based on the genomic location of the eight states.

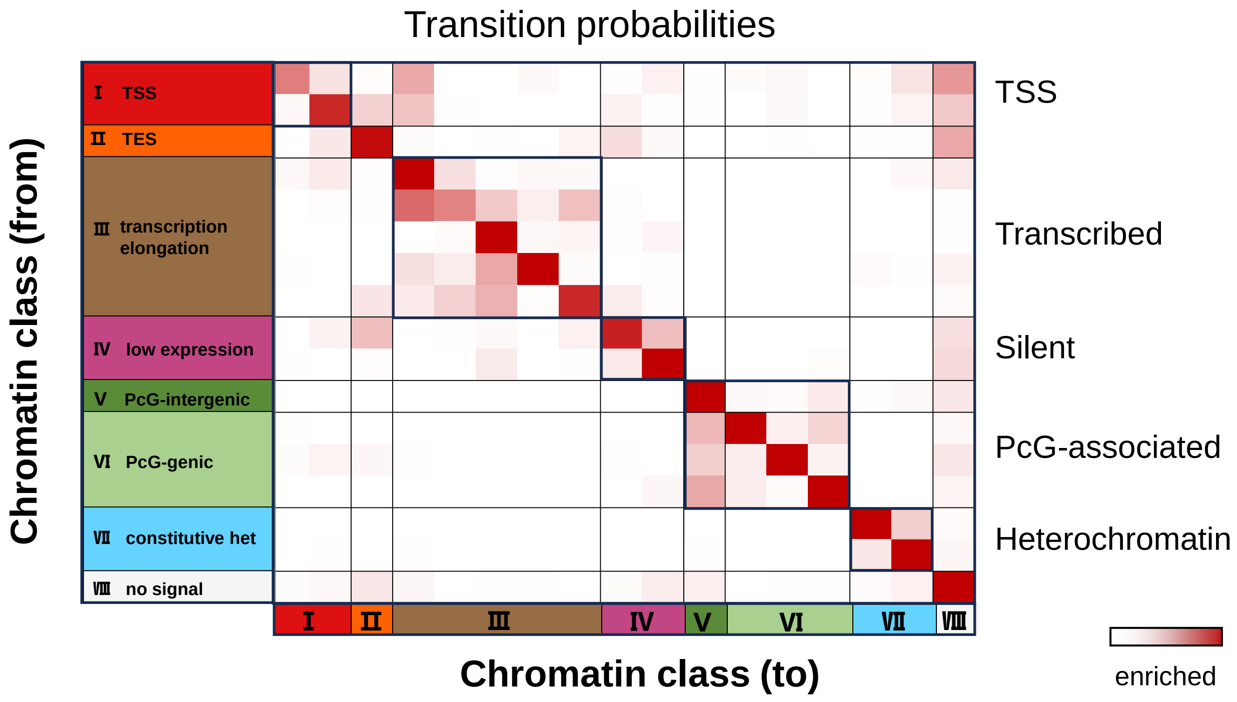

Fig. S3 The transition probabilities from one chromatin state (row) to another (column) in preT-stage and red-stage fruits. Values corresponds to transition probabilities from the class of the row to the class of the column were generated by chromHMM.

(A)

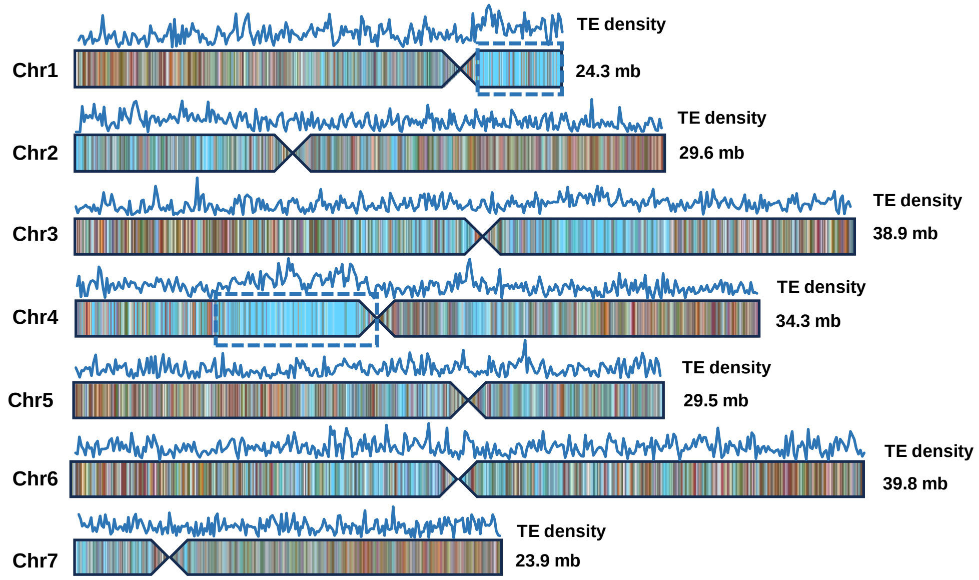

Fig. S4 Chromatin-centric annotation to the preT-stage fruits of *F. vesca*. A genome-wide *karyotype* view of the chromatin domains defined by the eight chromatin states in the preT-stage fruits of *F. vesca*. The eight chromatin states are described in Fig. S1. TE density is illustrated above each chromosome. Chromosomes 1 and 4 have broad H3K9me2-enriched domains which are indicative of pericentric heterochromatic regions.

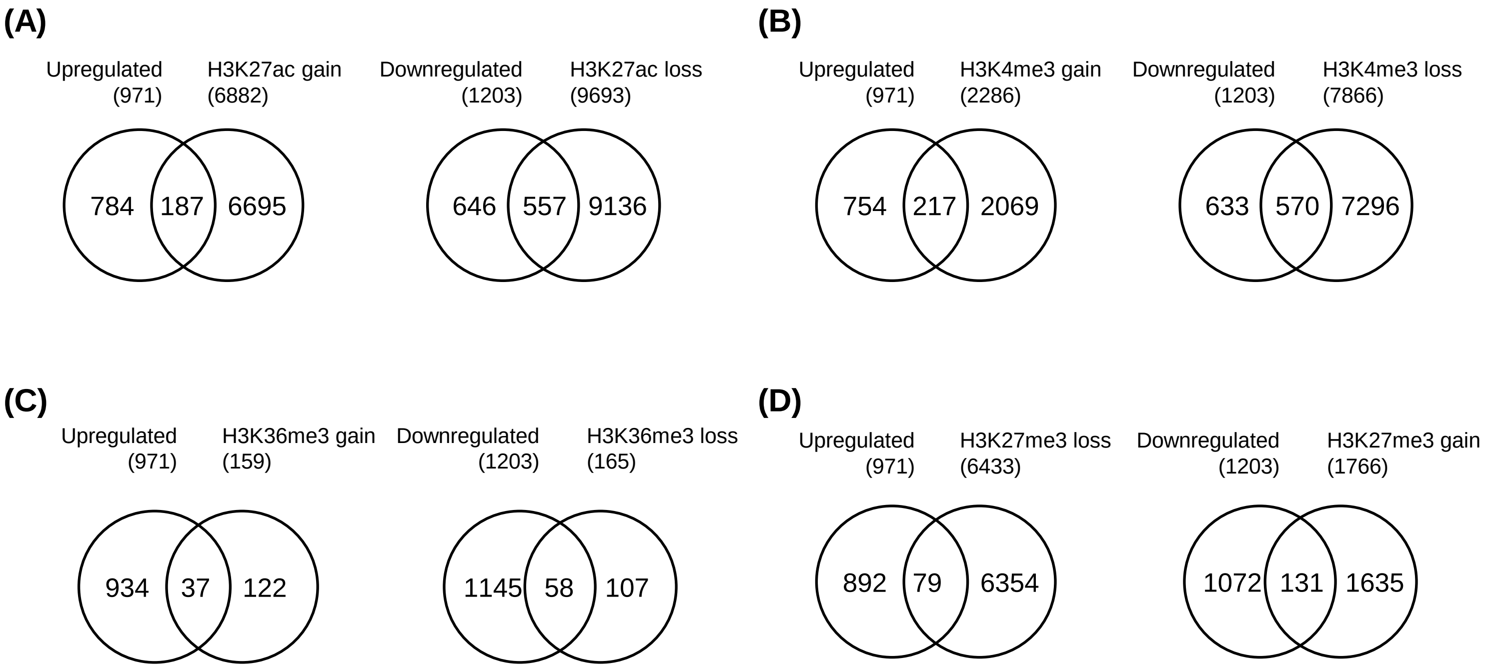

Fig. S5 Correlation between histone modification levels and expression levels of ripening-related genes. Venn diagrams illustrate the overlap between histone differentially peaks **(A**, H3K27ac; **B**, H3K4me3; **C**, H3K36me3; **D**, H3K27me3**)** and differentially expressed genes during ripening (preT-stage *vs*. red-stage. The gene body, 500 bp upstream of transcription start sites and 200 bp downstream of transcription end sites were considered. Histone differentially peaks, $|log_2FC| > 0.3$, FDR < 0.05; differentially expressed genes, FC >1.5, FDR < 0.05. preT, pre-turning; Data from *F. vesca* fruits.

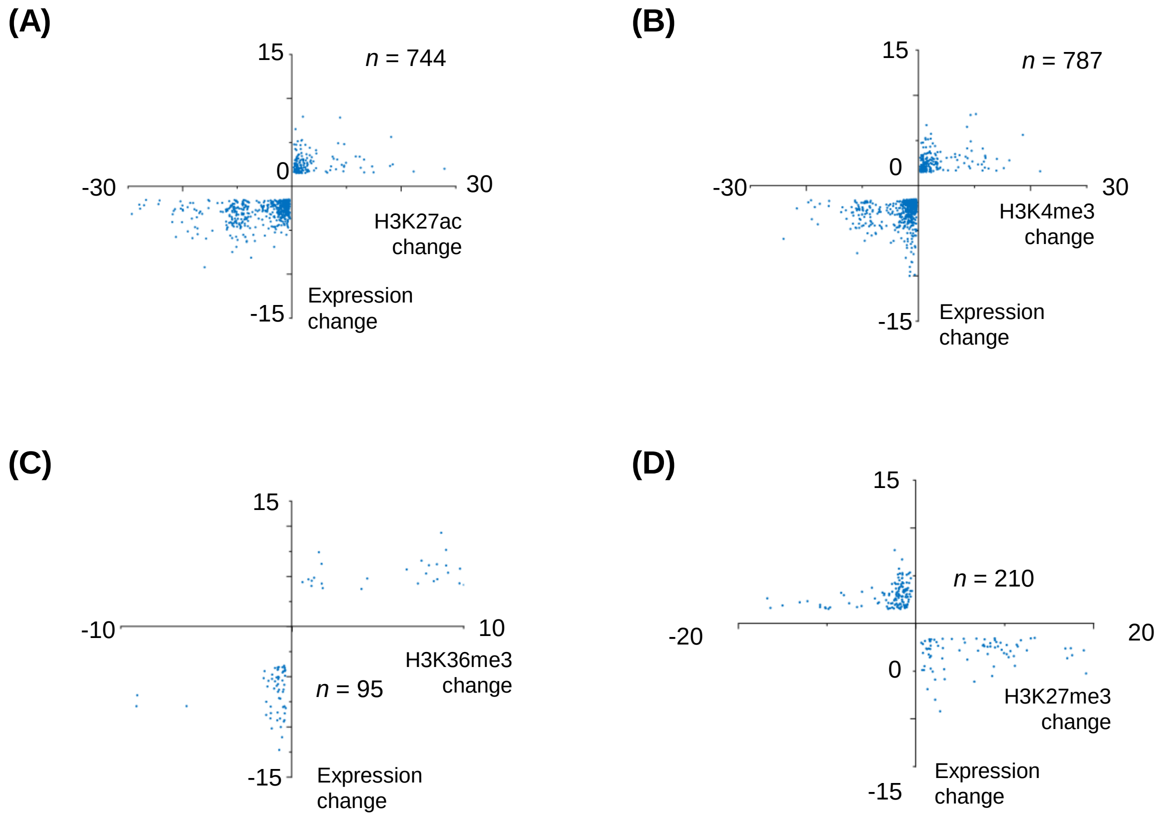

Fig. S6 The correlation between histone modifications changes and gene expression changes during ripening. Scatterplots display the correlation between histone modifications changes and gene expression changes during ripening. **(A**, H3K27ac; **B**, H3K4me3; **C**, H3K36me3; **D**, H3K27me3**)** The gene body, 500 bp upstream of transcription start sites and 200 bp downstream of transcription end sites were considered. Histone differentially peaks, $|\log_2 FC| > 0.3$, FDR < 0.05; differentially expressed genes, FC >1.5, FDR < 0.05. preT, pre-turning; Data from *F. vesca* fruits.

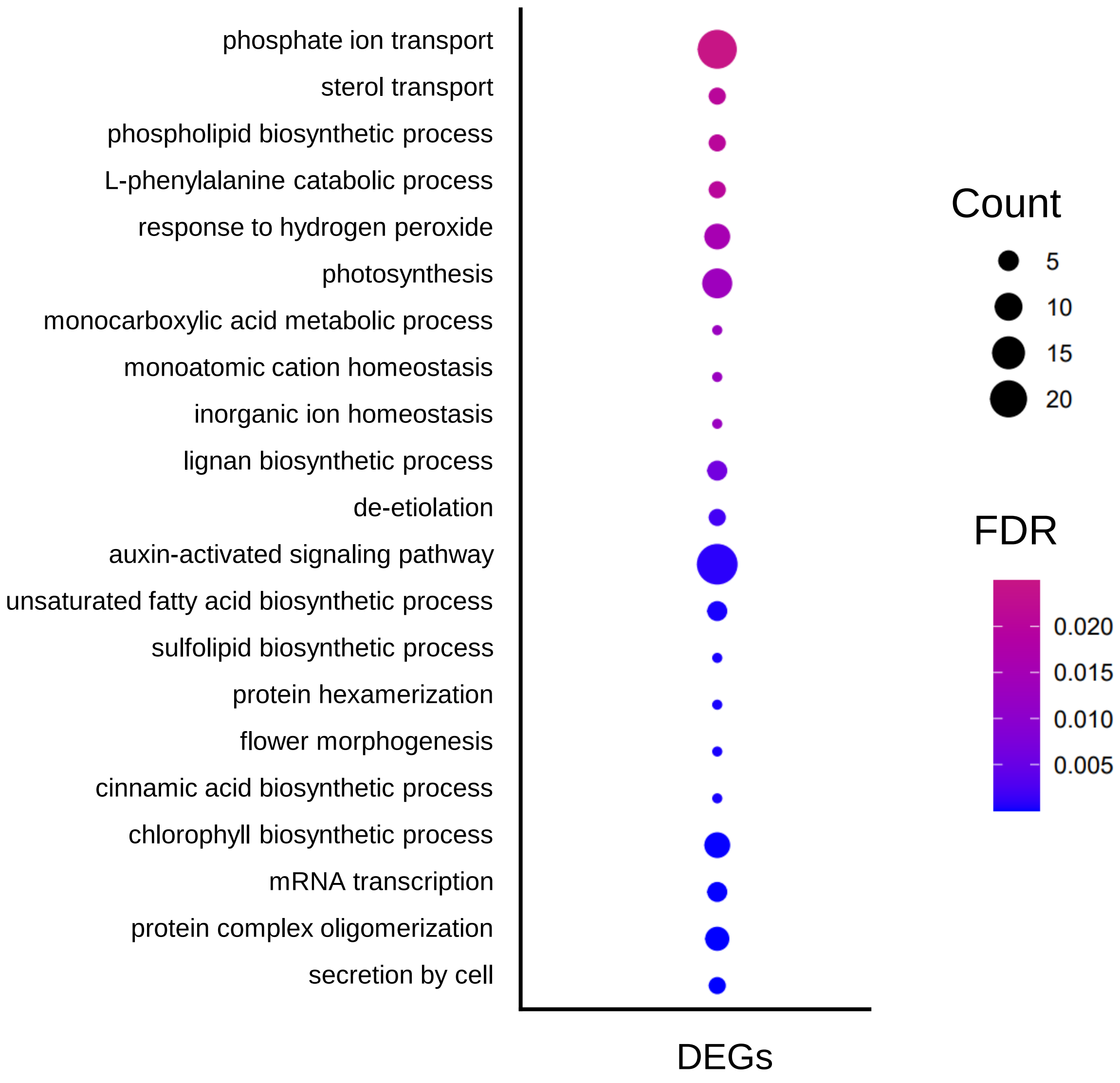

Fig. S7 Gene ontology enrichment analysis (biological process) of the differentially expressed genes during ripening (preT-stage *vs.* red-stage) which associated with significant H3K9/K14ac change.

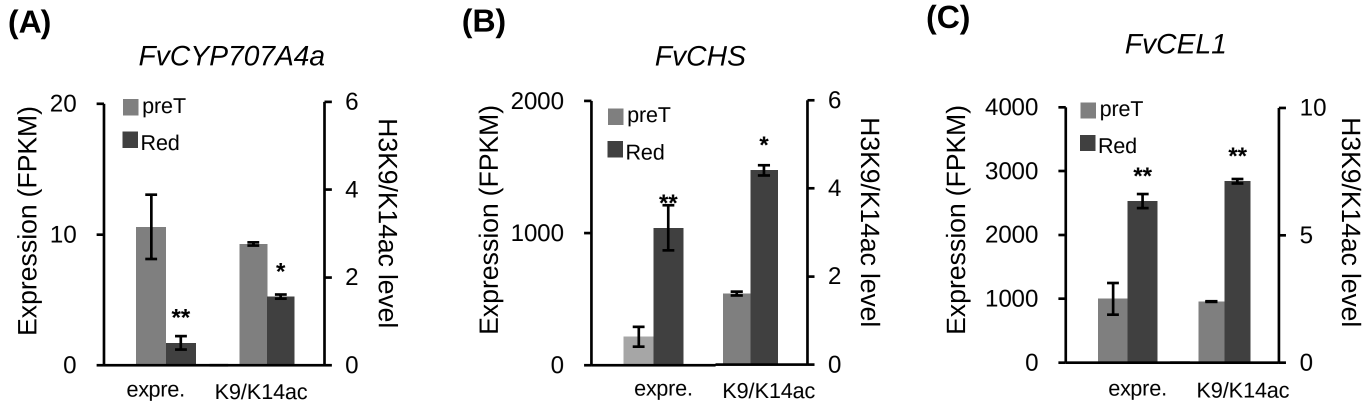

Fig. S8 Expression levels and H3K9/K14ac levels of some ripening-related genes. Bar graphs showing a positive correlation between expression levels (RNA-seq) and H3K9/K14ac levels (ChIP-seq) of the oxidative degradation gene *FvCYP707A4a* **(A)**, a chalcone synthase gene *FvCHS* **(B)**, and an endo-1,4-β-glucanase gene *FvCEL1* **(C)**. Read counts ratio (ChIP/Input) along the gene body was calculated as the H3K9/K14ac level for each sample. Error bars represent mean ± s.e.m. of $n = 3$ experiments (**A–C**, expression), or $n = 2$ experiments (**A–C**, H3K9/K14ac). * and ** denote statistical significance, FDR < 0.05 and < 0.01 **(A–C,** left**)**, $p < 0.05$ and < 0.01, student's *t*-test **(A–C**, right**)**. Histone differentially peaks, $|\log_2 FC| > 0.3$, FDR < 0.05; differentially expressed genes, FC >1.5, FDR < 0.05. preT, pre-turning. Data from *F. vesca* fruits.
